# Association between UMAP-identified body composition phenotypes and metabolic dysfunction-associated steatotic liver disease in the general population of China

**DOI:** 10.3389/fnut.2026.1873472

**Published:** 2026-08-03

**Authors:** Huimin Yin, Yangtian Wang, Li Yuan, Shuaiju Liao, Li Ding, Lili Liu, Dan Xiang

**Affiliations:** 1 Department of Clinical Nutrition, Taikang Xianlin Drum Tower Hospital, Affiliated Hospital of Medical School, Nanjing University, Nanjing, China; 2 Department of Endocrinology, Taikang Xianlin Drum Tower Hospital, Affiliated Hospital of Medical School, Nanjing University, Nanjing, China

**Keywords:** body composition phenotypes, cross-sectional association study, machine learning, metabolic dysfunction-associated steatotic liver disease (MASLD), UMAP

## Abstract

**Background:**

Metabolic dysfunction-associated steatotic liver disease (MASLD) poses a critical global health challenge, yet traditional anthropometric indices fail to precisely capture its metabolic heterogeneity. This study aimed to identify novel body composition phenotypes using nonlinear dimensionality reduction and to develop a predictive model for MASLD.

**Methods:**

A total of 7,946 participants were enrolled. Uniform Manifold Approximation and Projection (UMAP) was applied to bioelectrical impedance analysis (BIA) data for unsupervised clustering to identify latent phenotypes. Key variables were selected using a combination of Boruta, XGBoost, and Random Forest algorithms, followed by the construction of a Logistic regression model to evaluate associations and predictive performance.

**Results:**

UMAP robustly identified three distinct phenotypes: Low-Mass Lean (LML), High-Mass Obesogenic (HMO), and Fluid-Imbalanced, High-Parameters (FIHP). In terms of frequency, LML was the most common (47.7% in males, 49.2% in females), followed by HMO (45.8% and 44.8%, respectively) and the rare FIHP subtype (6.5% and 6.0%, respectively). LML exhibited normal size and metabolic balance, whereas HMO was characterized by high adiposity and metabolic risk, and FIHP by fluid imbalance. Compared to the LML group, HMO and FIHP phenotypes were associated with significantly higher risks of MASLD (adjusted OR: 3.10 [95% CI: 2.74–3.51] and 1.39 [95% CI: 1.08–1.78], respectively). The integrated model demonstrated superior predictive performance (AUC = 0.863) and favorable clinical utility in calibration and decision curve analyses.

**Conclusion:**

Our study identified three distinct body composition phenotypes significantly associated with MASLD risk using unsupervised clustering and demonstrated that a prediction model integrating these phenotypes with routine indicators exhibited superior performance, suggesting their potential utility for early risk stratification and clinical management.

## 1 Introduction

Metabolic dysfunction-associated steatotic liver disease (MASLD), formerly termed non-alcoholic fatty liver disease (NAFLD), has emerged as the leading cause of chronic liver disease worldwide, affecting an estimated 30% to 38% of the global adult population (1, 2). The surging prevalence of MASLD parallels rising rates of metabolic risk factors, particularly obesity, type 2 diabetes, and hypertension (3). Projections indicate further escalation fueled by aging populations and sedentary lifestyles, thereby exacerbating its impact as a critical global public health challenge (4). Driven by hepatic fat accumulation and systemic metabolic dysregulation, MASLD encompasses a pathological spectrum ranging from simple steatosis to metabolic dysfunction-associated steatohepatitis (MASH), with potential progression to fibrosis, cirrhosis, and hepatocellular carcinoma, imposing substantial clinical and economic burdens (5). Beyond the liver, MASLD is increasingly recognized as a multisystem disorder strongly associated with elevated risks of atherosclerotic cardiovascular disease (ASCVD), chronic kidney disease, and extrahepatic malignancies (6, 7).

The pathophysiology of MASLD is intimately linked to body composition (8). However, conventional anthropometric measures, such as body mass index (BMI), exhibit significant limitations in diagnostic and risk assessment accuracy, often failing to capture individual metabolic heterogeneity (9). Consequently, a paradigm shift is underway in clinical practice—moving from morphology-based metrics to composition-based approaches—to enable a more holistic and precise disease evaluation. In this context, bioelectrical impedance analysis (BIA) offers distinct advantages, providing a non-invasive, rapid, portable, and cost-effective assessment (10). By capturing multidimensional data on fat mass, skeletal muscle mass, body fluid distribution, and minerals, BIA provides a comprehensive reflection of the body’s metabolic status (11). Nevertheless, given the high-dimensional complexity of these parameters, traditional linear analytical methods are often inadequate for unraveling subtle underlying structures. Uniform Manifold Approximation and Projection (UMAP), as an emerging nonlinear dimensionality reduction technique, excels at preserving both local neighborhood structures and global topological relationships, thereby enabling the accurate identification of inherent clusters and revealing hidden subpopulations within body composition data (12).

Accordingly, this study aims to apply UMAP to multidimensional body composition data to identify distinct phenotypic clusters. We further investigate the association between these phenotypes and MASLD, and develop and validate a prediction model integrating body composition with routine physical examination indicators. Ultimately, this work seeks to provide novel evidence for the early clinical identification and risk stratification of MASLD.

## 2 Materials and methods

### 2.1 Study design and population

This study was designed as a retrospective cross-sectional study aimed at investigating the association between body composition indices and metabolic dysfunction-associated steatotic liver disease (MASLD). We included adult individuals who underwent health examinations at the Health Management Center of our hospital between January 1, 2023, and October 31, 2025.

The inclusion criteria were as follows: (1) age ≥ 18 years; (2) completion of a routine physical examination; and (3) availability of complete body composition analysis data.

Exclusion criteria included: (1) lack of hepatobiliary ultrasound examination results; and (2) diagnosis of secondary liver diseases, such as viral hepatitis, autoimmune liver disease, drug-induced liver injury, or hepatocellular carcinoma.

The study protocol adhered to the principles of the Declaration of Helsinki and was approved by the Ethics Review Committee of our hospital (Approval No. LS202319). Given the retrospective design and the use of anonymized data, the requirement for informed consent was waived.

### 2.2 Data collection and variable measurement

All data were systematically collected by professionals through the Hospital Information System (HIS) and Laboratory Information System (LIS).

Body composition measurement: Body composition was assessed using a direct segmental multi-frequency bioelectrical impedance analyzer [InBody 770, Korea (13)]. Participants were required to fast for at least 4 h, empty their bladder, and remove metal accessories prior to measurement. Daily instrument calibration was performed. Key anthropometric indicators included height, weight, body mass index (BMI), percentage of body fat (PBF), visceral fat area (VFA), skeletal muscle mass (SMM), skeletal muscle mass index (SMI), fat-free mass index (FFMI), fat mass index (FMI), waist circumference (WC), hip circumference (HC), and waist-to-hip ratio (WHR).

Covariate collection: Covariate data were derived from concurrent health examination records, including demographic characteristics (age, sex), vital signs (blood pressure, pulse), biochemical indices (liver and kidney function, lipid profile, glucose metabolism, thyroid function, tumor markers, and routine blood parameters), and medical history. Laboratory tests were performed using the Roche Cobas 8000 automatic biochemical analyzer.

### 2.3 Outcome definition: MASLD

The diagnosis of MASLD was based on the 2023 international expert consensus using a two-step approach (14). First, abdominal color Doppler ultrasound was performed by two experienced sonographers. Hepatic steatosis was diagnosed if at least two of the following sonographic features were present: (a) increased hepatic echogenicity compared to the renal cortex, (b) ultrasound beam attenuation, and (c) unclear visualization of intrahepatic vascular structures (15). In the presence of hepatic steatosis, participants were required to have at least one metabolic risk factor: (1) BMI ≥ 23 kg/m^2^ (Asian criteria); (2) previously diagnosed type 2 diabetes mellitus (T2DM) or meeting American Diabetes Association (ADA) diagnostic criteria (fasting plasma glucose [FPG] ≥ 7.0 mmol/L or HbA1c ≥ 6.5%); or (3) presence of at least two of the following risk factors: elevated blood pressure (BP ≥ 130/85 mmHg or history of hypertension), dyslipidemia (triglycerides [TG] ≥ 1.7 mmol/L, high-density lipoprotein cholesterol [HDL-C] < 1.0 mmol/L for males or < 1.3 mmol/L for females), or prediabetes (FPG 5.6–6.9 mmol/L or HbA1c 5.7%–6.4%).

### 2.4 Unsupervised cluster analysis

Data dimensionality reduction and cluster analysis were performed by combining Uniform Manifold Approximation and Projection (UMAP) with variance-maximizing rotation (16). First, all 27 body composition variables were standardized to zero mean and unit variance. UMAP parameters were optimized based on reconstruction fidelity; specifically, the n_neighbors value of 15 was chosen after evaluating Shepard Goodness and Trustworthiness across a range of values to balance local and global structure preservation (17). Optimization results for male and female cohorts are presented in Supplementary Figures 1, 2. The min_dist parameter was set to 0.1 to maintain intra-cluster cohesion without over-merging subpopulations (18). Dimensionality reduction was then conducted using Chebyshev distance with n_components = 2, n_neighbors = 15, and min_dist = 0.1. Subsequently, K-means clustering was applied to the dimension-reduced features. The optimal number of clusters was determined comprehensively using the elbow method combined with the number of peaks in the silhouette plot.

### 2.5 Statistical analysis

Statistical analyses were performed using Python 3.11.7 and R 4.3.2 software, with a *P*-value < 0.05 considered statistically significant.

Missing data for variables were imputed using Multiple Imputation by Chained Equations (MICE, *m* = 10). Details regarding missing data for variables were provided in the Supplementary Materials (Supplementary Table 1). Continuous variables with a normal distribution were presented as mean ± standard deviation (SD), while those with a skewed distribution were expressed as median (interquartile range 1, interquartile range 3). Differences between groups were analyzed using Analysis of Variance (ANOVA) or the Kruskal–Wallis H test, as appropriate. Categorical variables were expressed as frequencies and percentages, and intergroup differences were analyzed using the Chi-squared test.

Three machine learning algorithms—Boruta, XGBoost, and Random Forest (RF)—were applied in parallel to screen for key predictors. Covariate data were derived from concurrent health examination records, with specific details provided in Table 1. Hyperparameters for XGBoost and RF were optimized using fivefold cross-validation and random search. A normalized composite metric—the Ensemble Score—was computed by first converting each algorithm’s output into a comparable importance measure: Boruta’s Z-score, and the rank-based importance from XGBoost and RF (lower rank indicating higher importance), which were then min–max scaled per algorithm to ensure equal weighting; the final Ensemble Score was the arithmetic mean of these three normalized values. Features not selected by an algorithm received near-zero scores. The top 15 features based on comprehensive importance rankings were included in subsequent models. For regression analysis, multivariate logistic regression models were constructed to analyze the association between body composition and MASLD: Model 1 was unadjusted; Model 2 was adjusted for age and sex; and Model 3 was further adjusted for key covariates identified by machine learning. Results were expressed as odds ratios (OR) with 95% confidence intervals (CI). Additionally, subgroup analyses and interaction tests were performed stratified by sex, age (18–39, 40–59, ≥ 60 years), BMI, and disease status.

**TABLE 1:** Baseline characteristics of 7946 participants according to MASLD status.

Characteristic	Total (*N* = 7946)	NO- MASLD (*N* = 4784)	MASLD (*N* = 3162)	*P*	SMD
Male (*n*, %)	4500 (56.63)	2124 (44.40)	2376 (75.14)	< 0.001	0.660
Age (years)	51.38 ± 12.82	50.66 ± 13.33	52.47 ± 11.92	< 0.001	0.143
SBP (mmHg)	124.33 ± 17.48	120.88 ± 17.32	129.55 ± 16.39	< 0.001	0.514
DBP (mmHg)	73.83 ± 11.58	71.17 ± 11.09	77.86 ± 11.13	< 0.001	0.602
Pulse (bpm)	74.98 ± 10.54	74.36 ± 10.56	75.91 ± 10.44	< 0.001	0.147
BUN (mmol/L)	5.44 ± 1.34	5.34 ± 1.37	5.59 ± 1.27	< 0.001	0.185
Cr (μmol/L)	64.94 ± 15.50	62.81 ± 15.37	68.18 ± 15.12	< 0.001	0.352
UA (μmol/L)	341.02 ± 87.46	314.14 ± 79.16	381.69 ± 83.63	< 0.001	0.830
GFR (mL/min)	111.25 ± 22.33	111.88 ± 22.09	110.29 ± 22.66	0.002	0.071
ALT (U/L)	20.00 (15.00, 29.00)	17.00 (13.00, 23.00)	27.00 (20.00, 40.00)	< 0.001	0.617
AST (U/L)	21.00 (18.00, 25.00)	20.00 (17.00, 24.00)	23.00 (19.00, 28.00)	< 0.001	0.342
GGT (U/L)	24.00 (16.12, 40.00)	19.00 (14.00, 29.00)	35.00 (24.00, 54.00)	< 0.001	0.610
ALP (U/L)	72.58 ± 19.79	70.53 ± 19.87	75.69 ± 19.26	< 0.001	0.264
LDH (U/L)	177.88 ± 29.52	175.65 ± 27.98	181.24 ± 31.41	< 0.001	0.188
ChE (U/L)	386.00 (336.00, 444.00)	363.00 (320.00, 415.40)	418.30 (372.80, 471.45)	< 0.001	0.040
LAP (U/L)	51.18 ± 10.32	49.37 ± 9.51	53.92 ± 10.88	< 0.001	0.445
TP (g/L)	73.49 ± 4.08	73.27 ± 4.00	73.83 ± 4.16	< 0.001	0.136
ALB (g/L)	46.19 ± 2.50	46.01 ± 2.53	46.47 ± 2.44	< 0.001	0.182
GLOB (g/L)	27.30 ± 3.18	27.26 ± 3.14	27.36 ± 3.24	0.154	0.033
AGRatio	1.71 ± 0.21	1.71 ± 0.22	1.72 ± 0.21	0.059	0.043
TBIL (μmol/L)	12.00 (9.50, 15.20)	11.90 (9.40, 15.10)	12.16 (9.60, 15.40)	0.024	0.050
IBIL (μmol/L)	8.80 (7.00, 11.20)	8.80 (7.00, 11.10)	8.90 (7.10, 11.30)	0.066	0.047
DBIL (μmol/L)	3.14 (2.40, 4.10)	3.10 (2.40, 4.08)	3.20 (2.50, 4.10)	0.001	0.078
TBA (μmol/L)	3.00 (2.00, 4.70)	2.90 (1.90, 4.40)	3.30 (2.30, 5.10)	< 0.001	0.148
TC (mmol/L)	4.96 ± 0.99	4.94 ± 0.95	4.99 ± 1.05	0.028	0.051
TG (mmol/L)	1.31 (0.91, 1.99)	1.08 (0.77, 1.50)	1.84 (1.31, 2.67)	< 0.001	0.621
HDLC (mmol/L)	1.36 ± 0.35	1.47 ± 0.35	1.20 ± 0.28	< 0.001	0.859
LDLC (mmol/L)	3.12 ± 0.88	3.08 ± 0.87	3.17 ± 0.89	< 0.001	0.106
HbA1c (%)	5.86 ± 0.79	5.72 ± 0.63	6.09 ± 0.95	< 0.001	0.468
GLU (mmol/L)	5.43 ± 1.32	5.16 ± 0.99	5.85 ± 1.62	< 0.001	0.510
TSH (mIU/L)	2.09 (1.49, 2.85)	2.09 (1.49, 2.85)	2.09 (1.50, 2.84)	0.637	0.025
FT4 (pmol/L)	11.14 ± 1.98	11.20 ± 2.12	11.06 ± 1.75	0.002	0.072
FT3 (pmol/L)	5.39 ± 0.69	5.30 ± 0.71	5.54 ± 0.64	< 0.001	0.358
AFP (ng/mL)	3.09 (2.36, 4.12)	3.06 (2.31, 4.10)	3.14 (2.43, 4.13)	0.003	0.037
CEA (ng/mL)	1.68 (1.12, 2.41)	1.66 (1.11, 2.35)	1.73 (1.16, 2.51)	0.001	0.043
BMI (kg/m^2^)	24.68 ± 3.43	23.17 ± 2.75	26.97 ± 3.06	< 0.001	1.305
RBC (10∧12/L)	4.81 ± 0.49	4.70 ± 0.47	4.99 ± 0.47	< 0.001	0.621
HCT	43.87 ± 4.34	42.81 ± 4.28	45.47 ± 3.93	< 0.001	0.648
MCV (fL)	91.27 ± 4.70	91.24 ± 4.82	91.30 ± 4.50	0.584	0.013
HGB (g/L)	145.36 ± 15.69	141.33 ± 15.33	151.45 ± 14.20	< 0.001	0.685
MCH (pg)	30.23 ± 1.89	30.11 ± 1.94	30.41 ± 1.79	< 0.001	0.158
MCHC (g/L)	331.20 ± 9.89	329.99 ± 9.74	333.04 ± 9.84	< 0.001	0.312
WBC (10∧9/L)	5.97 ± 1.56	5.70 ± 1.47	6.37 ± 1.59	< 0.001	0.435
NeutPct (%)	56.99 ± 8.17	56.78 ± 8.50	57.31 ± 7.64	0.004	0.066
LymphPct (%)	34.41 ± 7.84	34.73 ± 8.14	33.93 ± 7.35	< 0.001	0.103
MonoPct (%)	5.71 ± 1.51	5.66 ± 1.52	5.78 ± 1.48	0.001	0.080
EoPct (%)	2.10 (1.30, 3.20)	2.00 (1.30, 3.10)	2.20 (1.50, 3.30)	< 0.001	0.095
BasoPct (%)	0.30 (0.20, 0.50)	0.30 (0.20, 0.50)	0.30 (0.20, 0.40)	< 0.001	0.150
NeutCount (10∧9/L)	3.26 (2.64, 4.04)	3.09 (2.51, 3.84)	3.54 (2.86, 4.33)	< 0.001	0.353
LymphCount (10∧9/L)	2.02 ± 0.61	1.94 ± 0.59	2.13 ± 0.61	< 0.001	0.311
BasoCount (10∧9/L)	0.02 (0.01, 0.03)	0.02 (0.01, 0.03)	0.02 (0.01, 0.03)	0.353	0.004
RDW_SD	13.11 ± 0.86	13.13 ± 0.92	13.07 ± 0.76	0.002	0.069
PLT (10∧9/L)	226.64 ± 56.19	227.24 ± 56.40	225.73 ± 55.87	0.240	0.027
MonoCount (10∧9/L)	0.32 (0.26, 0.40)	0.30 (0.24, 0.38)	0.35 (0.28, 0.43)	< 0.001	0.385
EoCount (10∧9/L)	0.12 (0.07, 0.19)	0.11 (0.07, 0.17)	0.14 (0.09, 0.21)	< 0.001	0.213
RDW_CV	44.12 ± 2.89	44.24 ± 2.91	43.94 ± 2.85	< 0.001	0.106
Hypertension (*n*, %)	2428 (30.56)	1072 (22.41)	1356 (42.88)	< 0.001	0.447
Diabetes (*n*, %)	1048 (13.19)	368 (7.69)	680 (21.51)	< 0.001	0.399
CHD (*n*, %)	123 (1.55)	61 (1.28)	62 (1.96)	0.015	0.054

AGRatio, albumin/globulin ratio; AFP, alpha-fetoprotein; ALB, albumin; ALP, alkaline phosphatase; ALT, alanine aminotransferase; AST, aspartate aminotransferase; BasoCount, basophil count; BasoPct, basophil percentage; BMI, body mass index; BUN, blood urea nitrogen; CEA, carcinoembryonic antigen; CHD, coronary heart disease; ChE, Cholinesterase; Cr, creatinine; DBIL, direct bilirubin; DBP, diastolic blood pressure; Diabetes, diabetes mellitus; EoCount, eosinophil count; EoPct, eosinophil percentage; FT3, free triiodothyronine; FT4, free thyroxine; GFR, glomerular filtration rate; GGT, gamma-glutamyl transferase; GLU, glucose; GLOB, globulin; HCT, hematocrit; HbA1c, hemoglobin A1c; HGB, hemoglobin; HDLC, high-density lipoprotein cholesterol; IBIL, indirect bilirubin; LAP, leucine aminopeptidase; LDH, lactate dehydrogenase; LDLC, low-density lipoprotein cholesterol; LymphCount, lymphocyte count; LymphPct, lymphocyte percentage; MCH, mean corpuscular hemoglobin; MCHC, mean corpuscular hemoglobin concentration; MCV, mean corpuscular volume; MonoCount, monocyte count; MonoPct, monocyte percentage; RBC, red blood cell; RDW_CV, red cell distribution width-coefficient of variation; RDW_SD, red cell distribution width-standard deviation; SBP, systolic blood pressure; TBA, total bile acid; TC, total cholesterol; TG, triglycerides; TSH, thyroid stimulating hormone; TBIL, total bilirubin; TP, total protein; UA, uric acid; WBC, white blood cell.

The specificity and sensitivity of the models were evaluated via Receiver Operating Characteristic (ROC) curve analysis and the corresponding Area Under the Curve (AUC). To ensure robust performance assessment, two complementary internal validation strategies were employed. First, hold-out validation was performed by randomly partitioning the dataset into a 70% training set and a 30% independent test set. Second, we applied bootstrap resampling (*B* = 300) to calculate optimism-corrected AUCs, thereby correcting for potential overfitting bias. Furthermore, decision curve analysis was conducted to assess the net benefit and clinical utility of the models.

## 3 Result

### 3.1 Baseline characteristics of the participants

Following the application of predefined inclusion and exclusion criteria, a final analytical sample of 7,946 participants was established, consisting of 4,784 individuals (60.21%) without MASLD (NO-MASLD group) and 3,162 patients (39.79%) with MASLD (MASLD group), as shown in Table 1. The overall mean age was 51.38 ± 12.82 years, with males comprising 56.63% of the study population. Compared to the NO-MASLD group (mean age: 50.66 ± 13.33 years; 44.40% male), the MASLD group was significantly older (mean age: 52.47 ± 11.92 years) and had a substantially higher proportion of males (75.14%, *P* < 0.001). Furthermore, patients with MASLD presented with a higher body mass index (BMI; 26.97 ± 3.06 kg/m^2^) than those without MASLD (23.17 ± 2.75 kg/m^2^). Consistent with the metabolic profile of the disease, the MASLD group exhibited elevated levels of systolic and diastolic blood pressure (SBP, DBP), blood urea nitrogen (BUN), creatinine (Cr), uric acid (UA), as well as liver enzymes (ALT, AST, GGT, ALP) and atherogenic lipids (TG, LDL-C), while HDL-C levels were significantly lower (all *P* < 0.001). Additionally, indices of glucose metabolism (HbA1c and GLU) were significantly elevated in the MASLD group. The prevalence of comorbidities, including hypertension (42.88% vs. 22.41%) and diabetes mellitus (21.51% vs. 7.69%), was also significantly higher in the MASLD group. No significant differences were observed in thyroid stimulating hormone (TSH) or platelets (PLT) levels between the groups (*P* > 0.05).

### 3.2 Identification of body composition clusters

**FIGURE 1:**
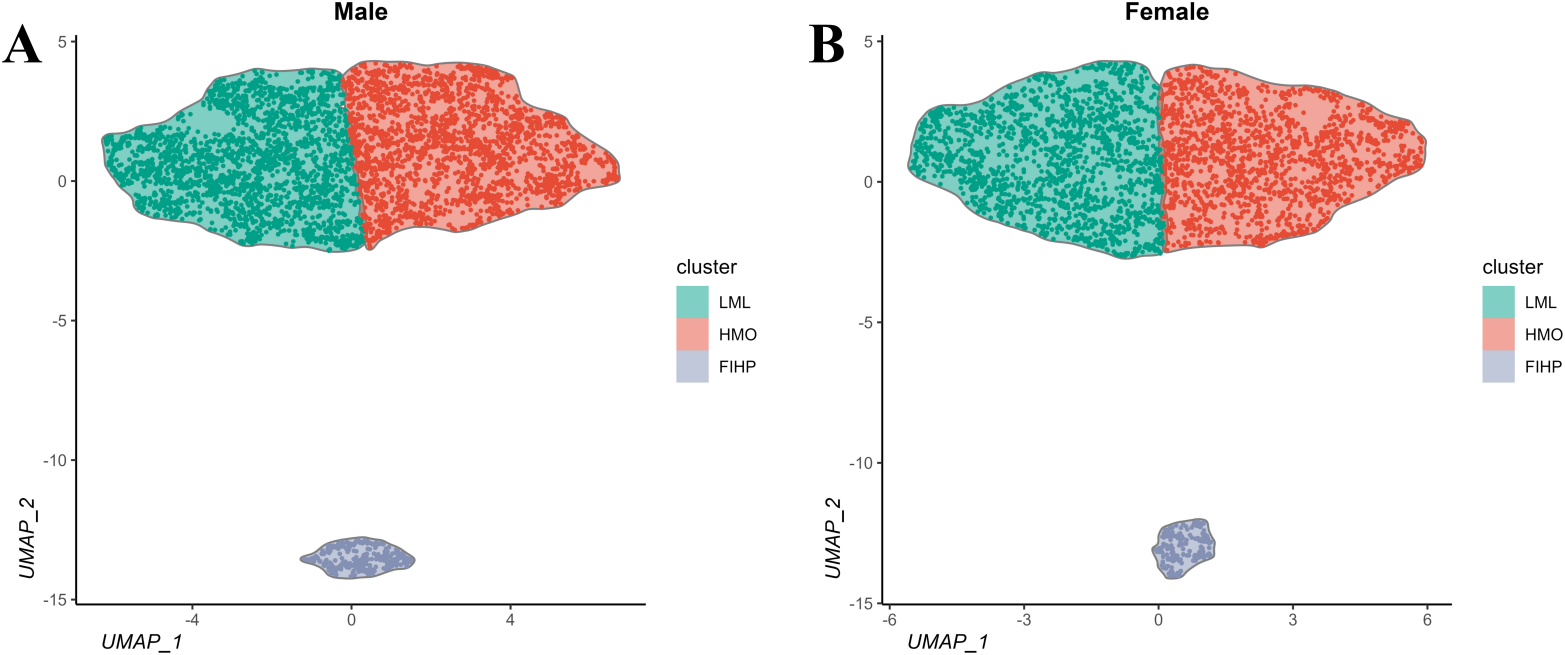
UMAP 2D projection of Body Composition Cluster Profiles in male **(A)** and female **(B)**. Colors denote profile allocations.

Gender-specific dimensionality reduction and clustering of 27 body composition variables were performed using Uniform Manifold Approximation and Projection (UMAP). The elbow method and silhouette analysis identified three as the optimal number of clusters. UMAP visualizations revealed consistent distribution patterns across genders (Figure 1). Cluster 1 (green area) represented the Low-Mass Lean (LML) group, Cluster 2 (red area) corresponded to the High-Mass Obesogenic (HMO) group, and Cluster 3 (blue area) denoted the Fluid-Imbalanced, High-Parameters (FIHP) group. The LML and HMO groups occupied the left and right sides of the plot, respectively, while the FIHP group was distinctly positioned at the bottom, indicating that the overall clustering structure was robust across genders. Among males, the distribution was 2,145 (47.7%) in LML, 2,062 (45.8%) in HMO, and 293 (6.5%) in FIHP. Among females, the subgroups comprised 1,694 (49.2%) LML, 1,546 (44.8%) HMO, and 206 (6.0%) FIHP. Baseline characteristics for each gender-specific cluster are detailed in the Supplementary Materials (Supplementary Tables 2, 3 and Supplementary Figure 3, 4).

### 3.3 Characteristics of body composition phenotypes

Analysis of the total sample (*n* = 7,946) revealed distinct body composition profiles across the three clusters (Table 2). The average participant had a height of 166.86 cm, weight of 69.10 kg, BMI of 24.68 kg/m^2^, and percent body fat (PBF) of 30.14%, classifying the study population as overweight according to WHO criteria.

**TABLE 2:** Characteristics of body composition clusters.

Characteristic	Total (*N* = 7946)	LML (*N* = 3839)	HMO (*N* = 3608)	FIHP (*N* = 499)	*P*	SMD
Male (*n*, %)	4500 (56.63)	2145 (55.87)	2062 (57.15)	293 (58.72)	0.337	0.038
Age (years)	51.38 ± 12.82	53.02 ± 13.00	50.77 ± 12.15	43.23 ± 12.60	< 0.001	0.518
Height (cm)	166.86 ± 8.37	164.48 ± 7.39	169.34 ± 8.55	167.24 ± 8.91	< 0.001	0.395
Weight (kg)	69.10 ± 13.04	61.75 ± 9.06	76.94 ± 11.97	68.99 ± 13.53	< 0.001	0.894
BMI (kg/m^2^)	24.68 ± 3.43	22.76 ± 2.52	26.75 ± 3.03	24.50 ± 3.48	< 0.001	0.897
TBW (L)	35.45 ± 7.15	32.40 ± 5.50	38.63 ± 7.28	35.91 ± 7.42	< 0.001	0.625
ICW (L)	21.13 ± 5.32	20.00 ± 3.49	23.91 ± 4.64	9.63 ± 2.04	< 0.001	2.857
ECW (L)	14.07 ± 3.52	12.39 ± 2.03	14.71 ± 2.66	22.29 ± 4.71	< 0.001	1.899
Protein Mass (kg)	9.73 ± 2.25	8.65 ± 1.51	10.34 ± 2.00	13.64 ± 2.73	< 0.001	1.528
Mineral Mass (kg)	3.28 ± 0.64	2.99 ± 0.45	3.58 ± 0.67	3.33 ± 0.68	< 0.001	0.667
Fat Mass (kg)	20.89 ± 6.28	17.72 ± 4.45	24.39 ± 6.12	20.05 ± 6.23	< 0.001	0.794
Muscle Mass (kg)	45.51 ± 9.23	41.58 ± 7.09	49.60 ± 9.40	46.15 ± 9.58	< 0.001	0.623
LBM (kg)	48.20 ± 9.75	44.04 ± 7.44	52.55 ± 9.93	48.87 ± 10.16	< 0.001	0.626
SMM (kg)	26.59 ± 5.92	24.09 ± 4.55	29.19 ± 6.04	27.05 ± 6.13	< 0.001	0.618
PBF (%)	30.14 ± 6.47	28.71 ± 5.97	31.83 ± 6.58	28.96 ± 6.55	< 0.001	0.325
Arm Muscle Balance (%)	0.06 (0.03, 0.10)	0.06 (0.03, 0.10)	0.07 (0.03, 0.11)	0.05 (0.02, 0.10)	< 0.001	0.119
Trunk Muscle Mass (kg)	21.98 ± 4.42	20.07 ± 3.52	24.00 ± 4.36	22.08 ± 4.53	< 0.001	0.640
Leg Muscle Balance (%)	0.07 (0.03, 0.13)	0.06 (0.03, 0.12)	0.08 (0.04, 0.14)	0.07 (0.03, 0.12)	< 0.001	0.121
VFA (cm^2^)	93.70 (73.20, 118.80)	79.20 (64.30, 97.30)	111.40 (91.77, 137.12)	88.00 (68.90, 110.60)	< 0.001	0.717
SMI (kg/m^2^)	7.17 ± 1.07	6.70 ± 0.91	7.65 ± 1.00	7.23 ± 1.09	< 0.001	0.645
FFMI (kg/m^2^)	17.14 ± 2.18	16.17 ± 1.78	18.16 ± 2.10	17.29 ± 2.22	< 0.001	0.661
FMI (kg/m^2^)	7.54 ± 2.30	6.59 ± 1.76	8.59 ± 2.36	7.21 ± 2.28	< 0.001	0.619
InBody score	68.58 ± 5.61	68.50 ± 4.90	68.53 ± 6.31	69.60 ± 5.28	< 0.001	0.136
BMC (kg)	2.73 ± 0.54	2.46 ± 0.36	2.95 ± 0.55	3.31 ± 0.50	< 0.001	1.235
TBW_LBM_Ratio	73.55 ± 0.24	73.57 ± 0.23	73.52 ± 0.25	73.61 ± 0.17	< 0.001	0.248
WC, (cm)	89.08 ± 10.29	83.36 ± 7.56	95.14 ± 9.34	89.29 ± 9.99	< 0.001	0.888
HC (cm)	96.54 ± 6.09	92.87 ± 4.35	100.27 ± 5.32	97.79 ± 5.82	< 0.001	0.976
WHR	0.92 ± 0.06	0.90 ± 0.05	0.95 ± 0.06	0.91 ± 0.06	< 0.001	0.632
Hypertension, (*n*, %)	2428 (30.56)	1029 (26.80)	1272 (35.25)	127 (25.45)	< 0.001	0.143
Diabetes (n, %)	1048 (13.19)	504 (13.13)	505 (14.00)	39 (7.82)	0.001	0.133
CHD (*n*, %)	123 (1.55)	59 (1.54)	62 (1.72)	2 (0.40)	0.082	0.086
MASLD (*n*, %)	3162 (39.79)	982 (25.58)	2000 (55.43)	180 (36.07)	< 0.001	0.421

BMC, bone mineral content; BMI, body mass index; CHD, coronary heart disease; ECW, extracellular water; FMI, fat mass index; FFMI, fat-free mass index; HC, hip circumference; ICW, intracellular water; LBM, lean body mass; PBF, percent body fat; SMI, skeletal muscle index; SMM, skeletal muscle mass; TBW, total body water; TBW_LBM_Ratio, ratio of total body water to lean body mass; VFA, visceral fat area; WC, waist circumference; WHR, waist-to-hip ratio.

The LML phenotype (*n* = 3,839 48.3%) was characterized by the lowest anthropometric measures, with an average BMI of 22.76 kg/m^2^ (normal range). This group exhibited the lowest adiposity profile, including fat mass (17.72 kg), fat mass index (FMI, 6.59 kg/m^2^), PBF (28.71%), and visceral fat area (VFA, median 79.20 cm^2^), as well as the lowest absolute lean mass (skeletal muscle mass [SMM] 24.09 kg; protein mass 8.65 kg; total body water [TBW] 32.40 L). Despite these lower absolute values, their InBody score (68.50) indicated a relatively balanced body composition.

The HMO phenotype (*n* = 3,608, 45.4%) represented the obesogenic end of the spectrum, displaying the highest anthropometric values (mean BMI 26.75 kg/m^2^) and adiposity indicators (fat mass 24.39 kg, PBF 31.83%, FMI 8.59 kg/m^2^, VFA median 111.40 cm^2^), suggestive of high metabolic risk. Although this phenotype possessed the highest absolute muscle and lean mass (SMM 29.19 kg, TBW 38.63 L), its InBody score (68.53) was representative of the intermediate cluster.

The FIHP phenotype (*n* = 499, 6.3%) presented with intermediate anthropometric measures (BMI 24.50 kg/m^2^) but was distinguished by severe fluid distribution anomalies, specifically extremely low intracellular water (ICW, mean 9.63 L) and high extracellular water (ECW, mean 22.29 L), alongside exceptionally high protein (13.64 kg) and bone mineral content (BMC 3.31 kg), indicating significant physiological disturbance. Given the severe ICW depletion, these elevated values are likely technical artifacts rather than true biological increases. Although muscle quality in this group may have been compromised by ICW depletion, the InBody score (69.60) was paradoxically the highest, driven by the influence of these aberrant readings on the scoring algorithm. Significant differences were observed for key variables across clusters (all *P* < 0.05), highlighting distinct physiological profiles.

### 3.4 Feature selection using machine learning

**FIGURE 2:**
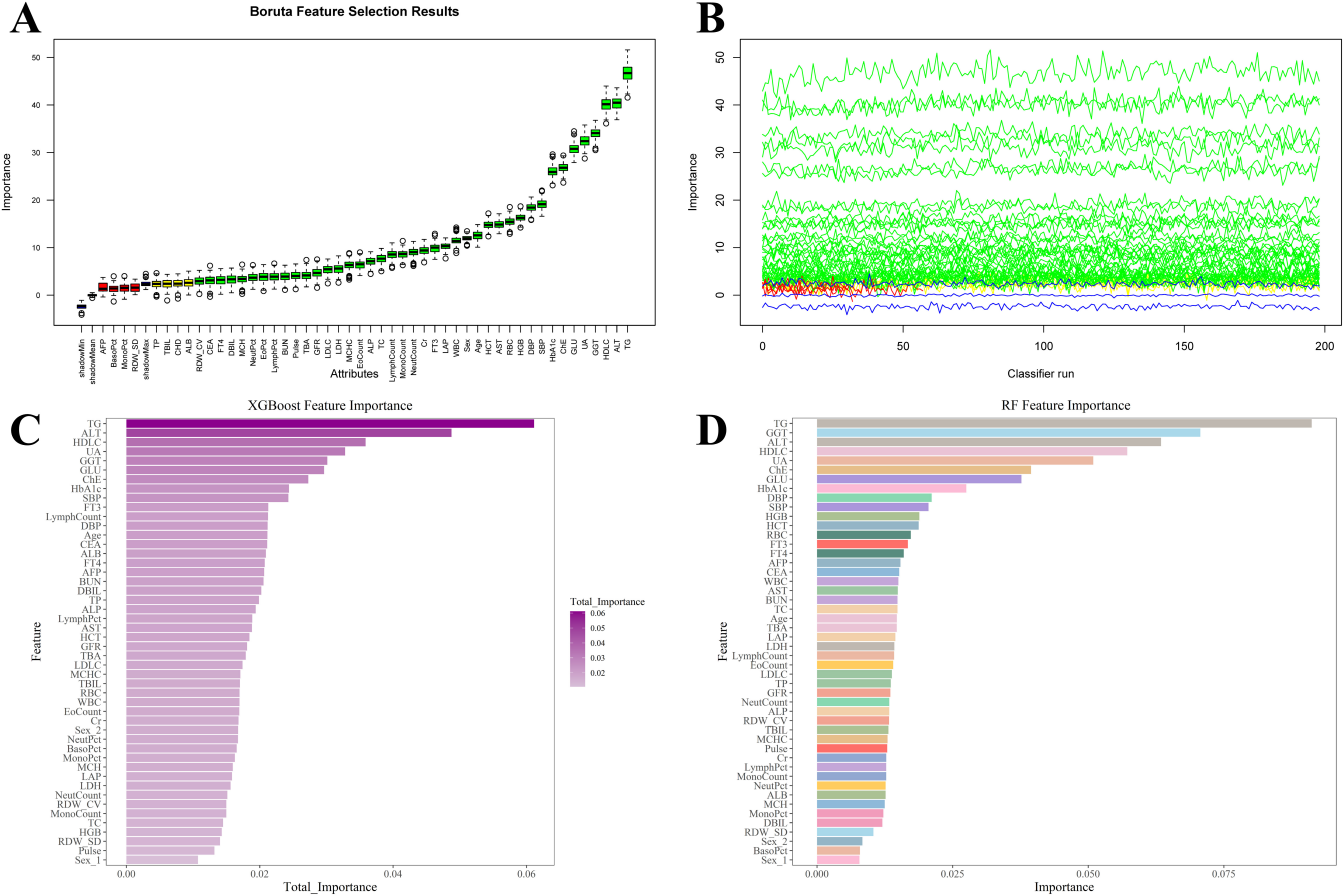
Feature selection results after computational processing using machine learning methods involving the Boruta **(A,B)**, XGBoost **(C)**, and RF **(D)** algorithms. **(A)** After 200 iterations of the Boruta algorithm, a subset of features was identified with the minimum cost function. The horizontal axis represents the input variables, and the vertical axis denotes feature importance (Z-scores). The blue boxes represent the minimum, average, and maximum Z-scores of shadow features, respectively. The green, yellow, and red boxes correspond to confirmed, tentative, and rejected features, respectively. **(B)** The iteration process of the Boruta algorithm. **(C)** All features ranked by importance based on the XGBoost algorithm. The lower the rank, the more important the variable. **(D)** All features ranked by the RF algorithm.

Three machine learning algorithms—Boruta, XGBoost, and Random Forest (RF)—were employed to identify key predictors of MASLD. The XGBoost and RF models were trained using fivefold cross-validation with hyperparameter optimization via random search. Features ranked within the top 15 in terms of importance were selected for subsequent model construction (Figures 2A–D). The final consensus on the top 15 critical features is presented in Supplementary Table 4. These variables—including TG, ALT, GGT, HDLC, UA, GLU, ChE, HbA1c, SBP, DBP, FT3, HCT, FT4, Age, and LymphCount—consistently demonstrated high importance across the different algorithms, all candidate predictors had VIF < 10 (Supplementary Table 5).

### 3.5 Association between body composition phenotypes and MASLD risk

Univariate and multivariate logistic regression analyses revealed that individuals in the HMO and FIHP groups had a significantly higher risk of MASLD compared to those in the LML group (Table 3). In the fully adjusted model (Model 3, Table 3), cluster assignment demonstrated a significant association with MASLD risk compared to both unadjusted and minimally adjusted models. Specifically, compared to the LML group, the HMO group showed an odds ratio (OR) of 3.62 (95% CI: 3.28–3.99) in Model 1, 4.46 (4.01–4.97) in Model 2, and 3.10 (2.74–3.51) in Model 3 (*P* < 0.001). Similarly, the FIHP group exhibited an OR of 1.64 (1.35–2.00) in Model 1, 2.05 (1.66–2.54) in Model 2, and 1.39 (1.08–1.78) in the fully adjusted Model 3 (*P* = 0.010).

**TABLE 3:** Multivariable logistic regression analyses for MASLD.

Variables	Model1		Model2		Model3	
	OR (95%CI)	*P*	OR (95%CI)	*P*	OR (95%CI)	*P*
Cluster						
LML	1.00 (Reference)	< 0.001	1.00 (Reference)	< 0.001	1.00 (Reference)	< 0.001
HMO	3.62 (3.28 ∼ 3.99)		4.46 (4.01 ∼ 4.97)		3.10 (2.74 ∼ 3.51)	
FIHP	1.64 (1.35 ∼ 2.00)	< 0.001	2.05 (1.66 ∼ 2.54)	< 0.001	1.39 (1.08 ∼ 1.78)	0.010

Model1: Crude; Model2: Adjust: Sex, Age; Model3: Adjust: Age, SBP, DBP, UA, ALT, GGT, ChE, TG, HDLC, HbA1c, GLU, FT4, FT3, HCT, LymphCount. ALT, alanine aminotransferase; ChE, cholinesterase; CI, confidence interval; DBP, diastolic blood pressure; FT3, free triiodothyronine; FT4, free thyroxine; GGT, gamma-glutamyl transferase; GLU, glucose; HbA1c, hemoglobin A1c; HCT, hematocrit; HDLC, high-density lipoprotein cholesterol; LDLC, low-density lipoprotein cholesterol; LymphCount, lymphocyte count; OR, odds ratio; SBP, systolic blood pressure; TG, triglycerides; UA, uric acid.

### 3.6 Predictive performance and clinical utility of the model

**FIGURE 3:**
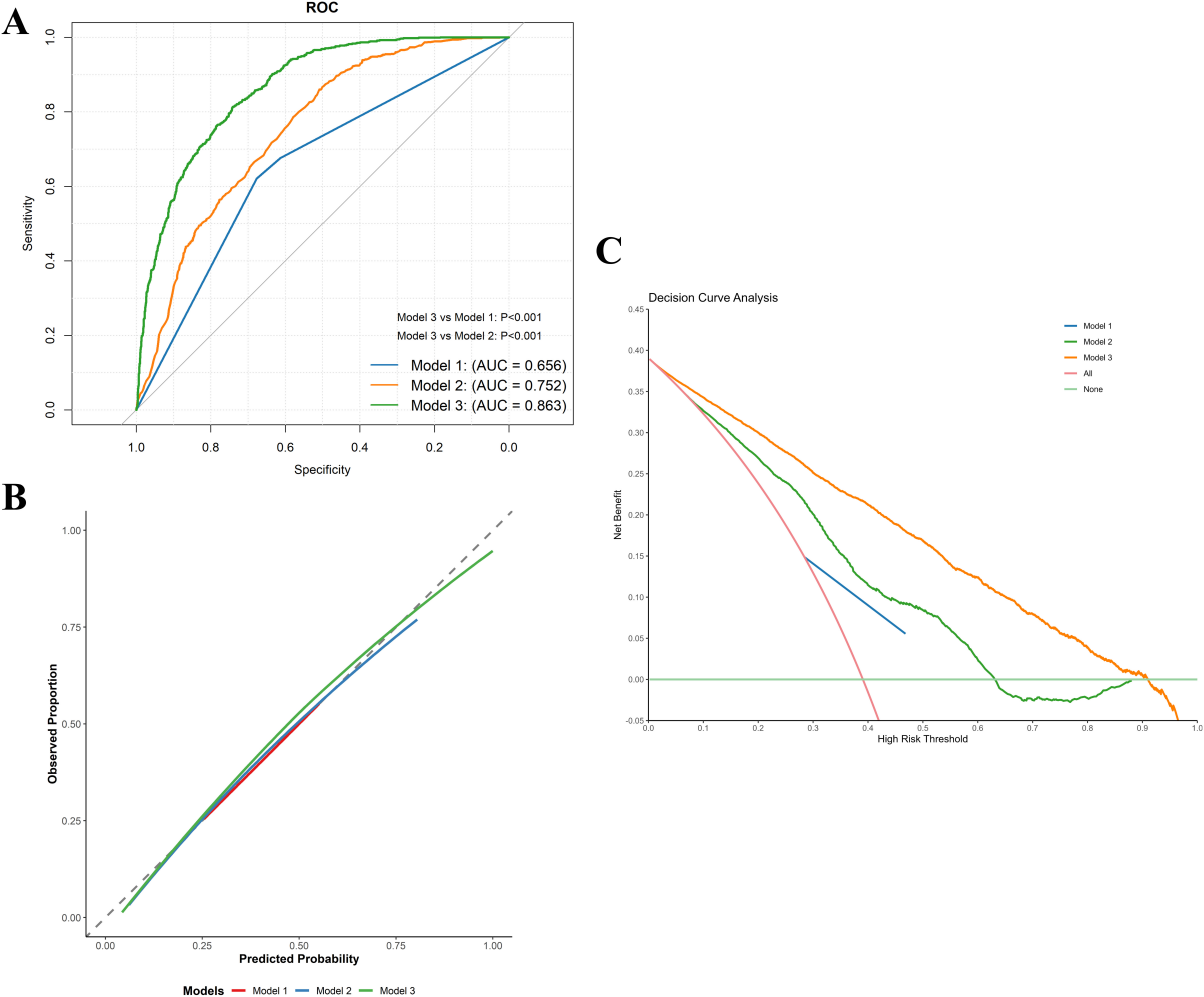
Performance evaluation of MASLD logistic regression models. **(A)** Receiver Operating Characteristic (ROC) Curves, **(B)** Calibration Curves Plots, **(C)** Decision Curve Analysis (DCA).

Receiver operating characteristic (ROC) curve analysis indicated that incorporating body composition phenotypes provided higher accuracy and discriminative power for MASLD prediction compared to the baseline model (Model 3 vs. Model 1: AUC = 0.863 vs. 0.656) after full adjustment (Figure 3A). DeLong’s test confirmed statistically significant differences in AUC values between the fully adjusted model and both the unadjusted and minimally adjusted models (*P* < 0.05). Internal validation confirmed robust performance: the hold-out AUC on the independent 30% test set was 0.863, and bootstrap optimism correction (*B* = 300) yielded a corrected AUC of 0.866, indicating minimal overfitting (optimism = 0.0012; Supplementary Table 6). Furthermore, calibration curves also showed the predictive probability of the fully adjusted model was roughly consistent with the actual probability (Figure 3B). Decision curve analysis (DCA) further revealed that the fully adjusted model offered a higher net benefit for predicting MASLD within a threshold probability range of 0 to 0.9 (Figure 3C).

### 3.7 Subgroup and sensitivity analyses

**FIGURE 4:**
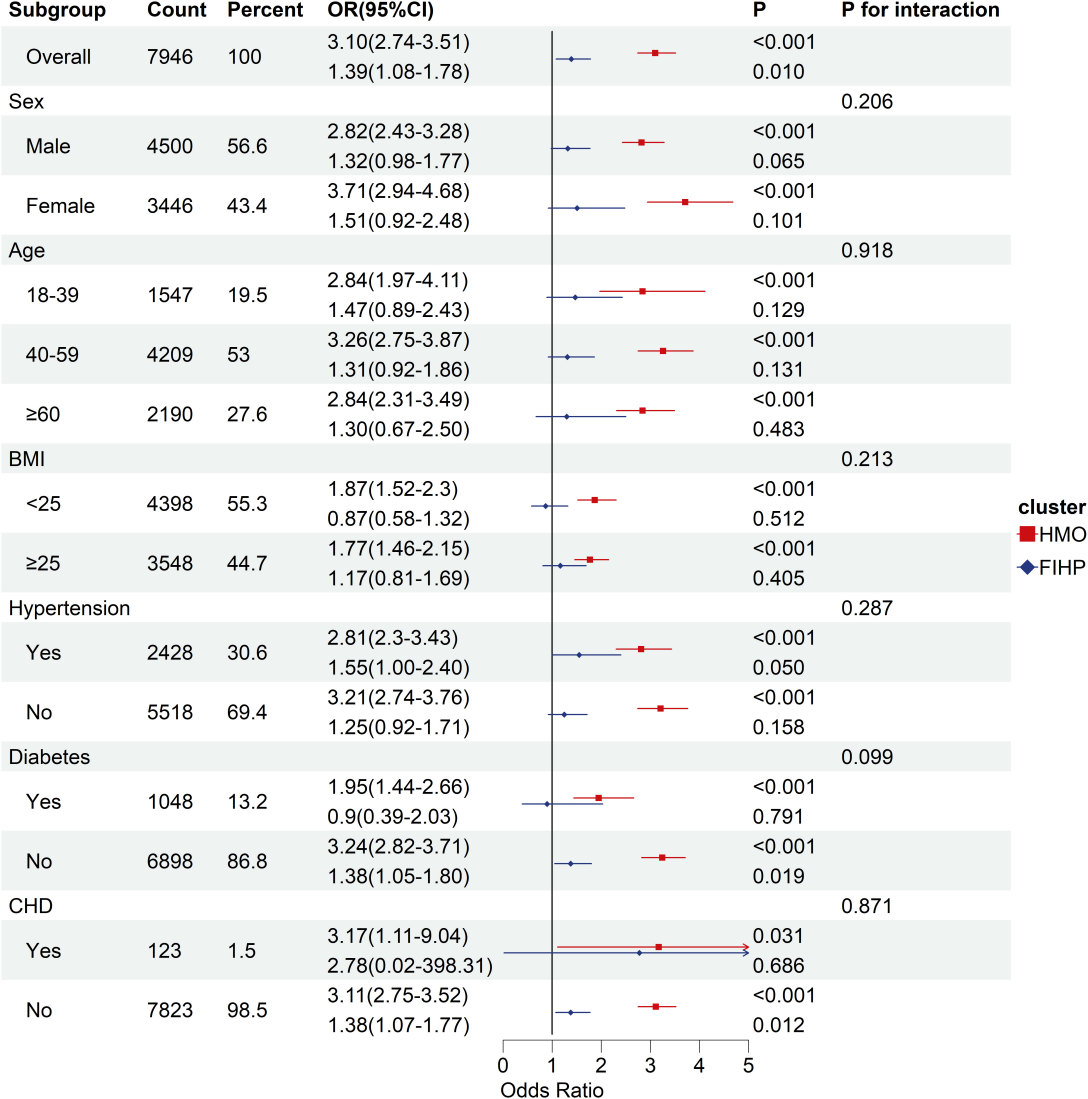
Subgroup analysis of the association between body composition clusters and MASLD. BMI, body mass index; CI, confidence interval; CHD, coronary heart disease; OR, odds ratio.

Subgroup and sensitivity analyses were conducted stratifying by gender, age, BMI, hypertension, diabetes, and coronary heart disease (CHD) status (Figure 4). Across all subgroups, the HMO phenotype consistently emerged as a significant risk factor for MASLD. Stratification by gender, age (18–39, 40–59, and ≥ 60 years), BMI ( < 25 kg/m^2^ and ≥ 25 kg/m^2^), hypertension, type 2 diabetes mellitus (T2DM), and CHD history did not significantly alter the association between metabolic phenotypes and MASLD (all interaction *P*-values > 0.05). Although the association between the FIHP phenotype and MASLD failed to reach statistical significance in certain subgroups (e.g., women, participants with BMI < 25 kg/m^2^, and patients with T2DM), the effect estimates remained elevated and in the same direction, and no evidence of effect modification was detected.

## 4 Discussion

In this large-scale cross-sectional analysis involving 7,946 participants, we were the first to apply Uniform Manifold Approximation and Projection (UMAP), a nonlinear dimensionality reduction technique, to perform unsupervised clustering on multiparameter body composition data derived from bioelectrical impedance analysis (BIA), and systematically evaluated the predictive value of the resulting body composition phenotypes for metabolic dysfunction-associated steatotic liver disease (MASLD). We demonstrated that UMAP robustly identified three discrete body composition phenotypes in both male and female populations: Low-Mass Lean (LML), High-Mass Obesogenic (HMO), and a novel phenotype characterized by fluid imbalance—Fluid-Imbalanced, High- Parameters (FIHP). Moreover, the HMO phenotype was significantly associated with MASLD risk compared to the LML phenotype; notably, this association remained robust even after adjusting for confounders such as age, sex, and blood pressure. Similarly, the FIHP phenotype also showed an independent increased risk. Furthermore, UMAP clustering variables combined with other physical examination indicators screened by three machine learning methods exhibited higher predictive performance for MASLD.

The escalating global disease burden of MASLD has created an urgent imperative for assessment tools that transcend traditional anthropometry to enable refined risk stratification (19). Conventional indicators, such as BMI, are widely utilized yet are characterized by significant limitations. Previous evidence indicates that BMI is notably deficient in predicting body fat distribution and mortality risk, often leading to the misclassification of individuals’ metabolic status (20). Specifically, BMI may fail to identify risks associated with normal-weight but centrally obese individuals, while simultaneously being unable to capture the protective metabolic effects conferred by high muscle mass (21). In contrast, BIA as a highly accessible, cost-effective, and reliable non-invasive method for body composition assessment, provides multidimensional quantitative parameters and demonstrates substantial concordance with gold-standard methods in MASLD evaluation (10, 22). However, the existing literature has predominantly focused on assessing the incremental predictive value of single indicators relative to BMI. For instance, analyses of 12 body composition indices suggest that while waist circumference or waist-to-height ratio may offer relatively superior predictive performance, such single-variable approaches remain insufficient for capturing the overall compositional characteristics and complex interactions of body composition (encompassing fat, muscle, and body fluids) (23). It is precisely this limitation that has driven a paradigm shift toward more comprehensive analytical methodologies. In recent years, machine learning techniques have been extensively applied to MASLD prediction, demonstrating robust performance. For example, a European study (24) utilized 19 routine clinical indicators to predict advanced outcomes (AUROC 0.719–0.994); a large-scale Chinese study (25) developed a model based on eight fundamental variables (internal AUROC 0.798–0.806, external 0.831); and a Korean study (26) further enhanced predictive efficacy by incorporating BIA parameters (achieving internal and external validation AUROCs of 0.90–0.91 and 0.88–0.89, respectively). Against this backdrop, the present study innovatively employs the UMAP algorithm. By performing comprehensive modeling on multi-parametric body composition data, this approach effectively overcomes the limitations of traditional single-indicator assessments and captures complex systemic metabolic patterns often missed by conventional features. The phenotypes identified herein are not merely simple extensions along a single dimension but rather holistic representations of the overall body composition status of the individual. Furthermore, by integrating routine physical examination indicators, we performed joint variable selection using three machine learning methods—BORUTA, XGBoost, and Random Forest. This process culminated in the inclusion of 15 indicators (encompassing age, demographic characteristics, metabolic indices, liver function, thyroid function, and complete blood count parameters) to construct a Logistic prediction model. This integrated model achieved an AUC of 0.868, effectively synthesizing multidimensional information and significantly enhancing the utilization efficiency of routine health checkup data, thereby providing a practical tool with tangible clinical implications for report interpretation, disease risk stratification, and early screening.

The identification of a novel FIHP phenotype is a key finding of this study, presenting a distinct MASLD risk profile. The key feature of this phenotype is significant abnormality in fluid distribution, specifically manifested as the relative expansion of extracellular water (ECW). The extracellular water/total body water (ECW/TBW) ratio measured by BIA has been confirmed to be a sensitive indicator for assessing systemic inflammatory status and tissue edema (27). In the field of hepatology, a cohort study has clearly demonstrated that an elevated ECW/TBW ratio correlates with the progression of liver fibrosis and serves as a predictive indicator for adverse clinical outcomes (28). Therefore, the fluid imbalance characteristics presented by the FIHP phenotype may mark a systemic low-grade inflammatory state (29). Although this state is not as intense as the obesity-inducing state manifested by the HMO phenotype, it is sufficient to cause continuous stress on liver metabolism by affecting microcirculation and exacerbating oxidative stress (30).

Furthermore, the high protein and mineral content accompanying this phenotype also deserves attention. Physiologically, given that protein mass is predominantly intracellular, the low ICW typically signifies muscle wasting rather than hypertrophy; thus, these findings likely reflect measurement artifacts induced by bioelectrical impedance analysis (BIA) under conditions of significant extracellular fluid expansion. Excessive ECW reduces overall impedance, causing standard algorithms to overestimate fat-free mass (FFM) and artifactually inflate calculated protein and mineral content. Consequently, the “high protein” status likely represents a pseudo-elevation driven by fluid retention rather than true biological status (26). This observed discrepancy also suggests potential limitations in the application of the InBody score within this study; as a proprietary composite index heavily weighted toward protein and minerals, the score may be erroneously elevated in the FIHP group, masking the underlying pathological state. Reliance on composite scores without scrutinizing raw hydration parameters may compromise the accuracy of clinical assessment in metabolically disturbed populations. In conclusion, the FIHP phenotype represents a group of occult risk populations easily overlooked by traditional BMI screening; BIA-derived fluid parameters provide a new perspective for identifying these “non-obese” high-risk individuals and establish potential intervention targets.

Subgroup analyses demonstrated that the HMO phenotype consistently acted as a high-risk factor for MASLD across diverse strata of gender, age, BMI, and comorbidities. In contrast, the newly identified FIHP phenotype, although confirmed as an independent risk factor for MASLD in the overall study population, demonstrated significantly attenuated associations in specific subgroups, such as females, individuals with BMI < 25 kg/m^2^, and patients with T2DM. Although the interaction *P*-values did not reach statistical significance, the robustness of the “High Adiposity-High Muscle Mass” pattern inherent to HMO implies that single-dimensional weight metrics may obscure potential metabolic risks. For instance, in gender-stratified analyses, the female HMO subgroup exhibited a numerically higher risk compared to males (OR: 3.71 vs. 2.82). More critically, among non-overweight individuals with BMI < 25 kg/m^2^, the HMO phenotype remained significantly associated with MASLD (OR: 1.87). This strongly supports the clinical concepts of “lean MASLD” or “normal weight metabolic obesity,” suggesting that reliance on BMI screening alone could result in missed diagnoses for a substantial subset of individuals with abnormal body composition but normal weight (31). Furthermore, in the subgroup with comorbid T2DM, the strength of the association for HMO was somewhat attenuated (OR: 1.95) but remained significant, likely constrained by the “saturation effect” resulting from severe insulin resistance and pre-existing hepatic steatosis (32). This further confirms that even in complex metabolic settings, optimizing overall body composition remains a key target for improving liver health (33). These findings underscore the translational value of integrating precise body composition assessments, such as BIA, into clinical practice. This integration not only facilitates the identification of high-risk HMO phenotypes for early screening in normal-weight individuals but also enables the formulation of differentiated intervention strategies based on the distinct pathological features of HMO and FIHP, thereby propelling MASLD management toward a more personalized and preventive model of care.

The intimate association between the HMO phenotype and MASLD may stem from the synergistic effects of visceral fat deposition, intramuscular fat infiltration, and a systemic low-grade inflammatory state, reflecting a state of metabolic overloading. Traditional views often regard obesity solely as the core of metabolic disturbances (34); however, accumulating evidence indicates that body composition quality is more critical than weight itself (26). Specifically, the HMO phenotype is defined not merely by an absolute expansion of fat mass but, more significantly, by pathological remodeling of non-adipose tissues. While skeletal muscle is typically viewed as a metabolically protective organ, the elevated skeletal muscle index (SMI) associated with the HMO phenotype is paradoxically accompanied by deteriorating intramuscular fat infiltration (35). Indeed, Wan et al. (36) established a positive correlation between SMI, visceral fat indices, and the severity of pathological hepatic steatosis in obese adults. Moreover, a cross-sectional study indicated that in the context of so-called “obesity-type sarcopenia,” the coexistence of high visceral fat—despite potentially high absolute SMI values—confers a significantly elevated risk of MASLD (37). Concurrently, the FIHP phenotype identified in this study may point to early pathological processes involving subclinical inflammation and tissue remodeling, representing a pathogenic pathway distinct from HMO. An elevated ECW/TBW ratio in BIA is closely associated with systemic inflammation and oxidative stress (29). Such fluid imbalance is frequently accompanied by impaired cell membrane integrity, thereby potentially increasing the liver’s susceptibility to metabolic injury (28). Consequently, the HMO and FIHP phenotypes may represent distinct pathogenic mechanisms: the former primarily reflects the metabolic burden associated with overload driven by adiposity, while the latter foreshadows microenvironmental changes characterized by tissue edema and a pro-fibrotic tendency.

The primary strengths of this study lie in its innovative design, large sample size, and the identification of a novel body composition phenotype with significant clinical implications. Nevertheless, several limitations should be acknowledged. First, the cross-sectional design precludes causal inferences. The observed associations, particularly for the FIHP phenotype, may be subject to reverse causality, where MASLD-related inflammation alters body composition. Second, MASLD was diagnosed via ultrasonography rather than biopsy, limiting sensitivity for mild steatosis. Potential diagnostic misclassification may be further compounded by the hydration-dependent nature of BIA. Third, BIA has inherent methodological constraints; factors like hydration status may compromise accuracy, suggesting that the Fluid-Imbalanced, High-Parameters (FIHP) phenotype could reflect measurement artifacts or edema rather than a distinct biological entity, necessitating validation via CT or DEXA. Fourth, the lack of detailed dietary data restricts the analysis of nutritional drivers. Additionally, BMI-based stratification may not fully capture metabolic risk compared to body composition-specific metrics. Finally, the generalizability of the findings is limited to populations with similar racial and demographic characteristics.

## 5 Conclusion

In conclusion, this comprehensive cross-sectional study leverages an innovative unsupervised clustering approach to integrate multidimensional body composition data for the first time, identifying three distinct phenotypes significantly associated with the risk of MASLD: Low-Mass Lean (LML), High-Mass Obesogenic (HMO), and Fluid-Imbalanced, High-Parameters (FIHP). Notably, the FIHP phenotype, characterized by moderate risk, may be closely linked to fluid imbalance and subclinical inflammation. Our findings elucidate the critical association between these body composition phenotypes and MASLD risk, underscoring the substantial value of routine body composition analysis in disease risk stratification. In addition, the body composition phenotypes combined with other physical examination indicators screened by three machine learning methods exhibited higher predictive performance for MASLD. Consequently, we recommend that clinicians incorporate body composition-related metrics into routine patient management to facilitate more precise and effective interventions.

## Data Availability

The datasets presented in this article are not readily available because data are restricted for analysis use only by personnel of this medical institution. Requests to access the datasets should be directed to the corresponding author.
