# Supplementary material for: Association between UMAP-identified body composition phenotypes and metabolic dysfunction-associated steatotic liver disease in the general population of China
Source: Front Nutr. 2026 Aug 3;13:1873472. doi: 10.3389/fnut.2026.1873472 (PMC13479794; doi:10.3389/fnut.2026.1873472)
Supplement: Supplementary file 1 [file Table_1.docx]

**Supplementary** **material**
Table S1. Proportion of missing values by variable in the baseline cohort (N = 7,946)

| Characteristic | Missing n (%) | Characteristic | Missing n (%) |
| --- | --- | --- | --- |
| ChE (U/L) | 1405 (17.682) | ALT (U/L) | 10 (0.126) |
| LAP (U/L) | 1405 (17.682) | AST (U/L) | 10 (0.126) |
| GGT (U/L) | 1400 (17.619) | GLU (mmol/L) | 11 (0.138) |
| LDH (U/L) | 1322 (16.635) | AFP (ng/mL) | 54 (0.680) |
| HbA1c (%) | 1364 (17.166) | TP (g/L) | 37 (0.466) |
| ALP (U/L) | 1282 (16.134) | ALB (g/L) | 37 (0.466) |
| IBIL (μmol/L) | 1283 (16.146) | GLOB (g/L) | 37 (0.466) |
| DBIL (μmol/L) | 1280 (16.109) | AGRatio | 37 (0.466) |
| FT3 (pmol/L) | 1112 (13.994) | TBIL (μmol/L) | 35 (0.440) |
| FT4 (pmol/L) | 1112 (13.993) | TC (mmol/L) | 35 (0.440) |
| TSH (mIU/L) | 1111 (13.982) | TG (mmol/L) | 35 (0.440) |
| RDW_CV | 157 (1.976) | HDLC (mmol/L) | 44 (0.554) |
| CEA (ng/mL) | 163 (2.051) | LDLC (mmol/L) | 44 (0.554) |
| TBA (μmol/L) | 172 (2.164) | InBody Score | 3 (0.038) |
| BasoCount (10⁹/L) | 145 (1.825) | BMC (kg) | 499 (6.280) |
| BasoPct (%) | 145 (1.825) | WC (cm) | 499 (6.280) |
| EoCount (10⁹/L) | 145 (1.825) | TBW_LBM_Ratio | 499 (6.280) |
| EoPct (%) | 145 (1.825) | Sex | 0 (0.000) |
| LymphCount (10⁹/L) | 145 (1.825) | Age (years) | 0 (0.000) |
| LymphPct (%) | 145 (1.825) | Height (cm) | 0 (0.000) |
| MonoCount (10⁹/L) | 145 (1.825) | Weight (kg) | 0 (0.000) |
| MonoPct (%) | 145 (1.825) | BMI (kg/m²) | 0 (0.000) |
| NeutCount (10⁹/L) | 145 (1.825) | TBW (L) | 0 (0.000) |
| NeutPct (%) | 145 (1.825) | ICW (L) | 0 (0.000) |
| RBC (10¹²/L) | 145 (1.825) | ECW (L) | 0 (0.000) |
| HCT | 145 (1.825) | Protein Mass (kg) | 0 (0.000) |
| MCV (fL) | 145 (1.825) | Mineral Mass (kg) | 0 (0.000) |
| HGB (g/L) | 145 (1.825) | Fat Mass (kg) | 0 (0.000) |
| MCH (pg) | 145 (1.825) | Muscle Mass (kg) | 0 (0.000) |
| MCHC (g/L) | 145 (1.825) | LBM (kg) | 0 (0.000) |
| WBC (10⁹/L) | 145 (1.825) | SMM (kg) | 0 (0.000) |
| PLT (10⁹/L) | 145 (1.825) | PBF (%) | 0 (0.000) |
| RDW_SD | 145 (1.825) | Arm Muscle Balance (%) | 0 (0.000) |
| SBP (mmHg) | 114 (1.435) | Trunk Muscle Mass (kg) | 0 (0.000) |
| DBP (mmHg) | 114 (1.435) | Leg Muscle Balance (%) | 0 (0.000) |
| Pulse (bpm) | 114 (1.435) | VFA (cm²) | 0 (0.000) |
| BUN (mmol/L) | 12 (0.151) | SMI (kg/m²) | 0 (0.000) |
| Cr (μmol/L) | 12 (0.151) | FFMI (kg/m²) | 0 (0.000) |
| UA (μmol/L) | 12 (0.151) | FMI (kg/m²) | 0 (0.000) |
| GFR (mL/min) | 12 (0.151) |  |  |

Abbreviations: AGRatio, albumin/globulin ratio; AFP, alpha-fetoprotein; ALB, albumin; ALP, alkaline phosphatase; ALT, alanine aminotransferase; AST, aspartate aminotransferase; BasoCount, basophil count; BasoPct, basophil percentage; BMC, bone mineral content; BMI, body mass index; BUN, blood urea nitrogen; CEA, carcinoembryonic antigen; CHD, coronary heart disease; ChE, cholinesterase; Cr, creatinine; DBIL, direct bilirubin; DBP, diastolic blood pressure; Diabetes, diabetes mellitus; EoCount, eosinophil count; EoPct, eosinophil percentage; ECW, extracellular water; FFMI, fat-free mass index; FC, not used (removed); FT3, free triiodothyronine; FT4, free thyroxine; GFR, glomerular filtration rate; GGT, gamma-glutamyl transferase; GLU, glucose; GLOB, globulin; HCT, hematocrit; HbA1c, hemoglobin A1c; HGB, hemoglobin; HDLC, high-density lipoprotein cholesterol; HC, hip circumference; IBIL, indirect bilirubin; ICW, intracellular water; LAP, leucine aminopeptidase; LDH, lactate dehydrogenase; LDLC, low-density lipoprotein cholesterol; LBM, lean body mass; Leg Muscle Balance, leg muscle balance; Lipid, not used (removed); LymphCount, lymphocyte count; LymphPct, lymphocyte percentage; MCH, mean corpuscular hemoglobin; MCHC, mean corpuscular hemoglobin concentration; MCV, mean corpuscular volume; MonoCount, monocyte count; MonoPct, monocyte percentage; PBF, percent body fat; PLT, platelet count; RBC, red blood cell; RDW_CV, red cell distribution width-coefficient of variation; RDW_SD, red cell distribution width-standard deviation; SBP, systolic blood pressure; SMI, skeletal muscle index; SMM, skeletal muscle mass; TBA, total bile acid; TC, total cholesterol; TG, triglycerides; TP, total protein; TSH, thyroid stimulating hormone; TBIL, total bilirubin; TBW, total body water; TBW_LBM_Ratio, ratio of total body water to lean body mass; UA, uric acid; VFA, visceral fat area; WC, waist circumference; WHR, waist-to-hip ratio; WBC, white blood cell.

Table S2. Characteristics of Body Composition Cluster Profiles in males.

| Characteristic | Total (*N* =4500) | LML (*N* =2145) | HMO (*N* =2062) | FIHP(*N* =293) | *P* | SMD |  |
| --- | --- | --- | --- | --- | --- | --- | --- |
| Age (years) | 51.44±12.94 | 54.60±12.72 | 49.37±12.21 | 42.87±12.79 | <0.001 | 0.620 |  |
| Height (cm) | 172.10±6.06 | 169.14±5.20 | 175.12±5.27 | 172.55±6.49 | <0.001 | 0.720 |  |
| Weight (kg) | 76.18±11.27 | 68.19±6.11 | 84.53±9.07 | 75.94±11.95 | <0.001 | 1.247 |  |
| BMI (kg/m²) | 25.68±3.22 | 23.88±2.31 | 27.59±2.92 | 25.45±3.29 | <0.001 | 0.884 |  |
| TBW (L) | 40.46±5.00 | 36.74±2.78 | 44.27±3.69 | 40.84±5.29 | <0.001 | 1.342 |  |
| ICW (L) | 24.17±4.64 | 22.76±1.77 | 27.51±2.34 | 11.00±1.43 | <0.001 | 6.037 |  |
| ECW (L) | 16.00±3.19 | 13.98±1.05 | 16.75±1.40 | 25.44±3.32 | <0.001 | 3.434 |  |
| Protein Mass (kg) | 11.15±1.81 | 9.84±0.77 | 11.89±1.02 | 15.44±1.97 | <0.001 | 2.761 |  |
| Mineral Mass (kg) | 3.69±0.52 | 3.32±0.28 | 4.07±0.41 | 3.73±0.56 | <0.001 | 1.253 |  |
| Fat Mass (kg) | 21.18±6.49 | 18.29±4.80 | 24.30±6.60 | 20.36±6.44 | <0.001 | 0.670 |  |
| Muscle Mass (kg) | 51.98±6.44 | 47.18±3.58 | 56.88±4.76 | 52.55±6.79 | <0.001 | 1.344 |  |
| LBM (kg) | 55.00±6.86 | 49.90±3.80 | 60.23±5.08 | 55.61±7.24 | <0.001 | 1.343 |  |
| SMM (kg) | 30.75±4.12 | 27.68±2.31 | 33.88±3.05 | 31.15±4.35 | <0.001 | 1.337 |  |
| PBF (%) | 27.38±5.46 | 26.54±5.39 | 28.41±5.34 | 26.32±5.51 | <0.001 | 0.259 |  |
| Arm Muscle Balance (%) | 0.08 (0.04, 0.13) | 0.08 (0.04, 0.12) | 0.08 (0.04, 0.14) | 0.07 (0.03, 0.13) | <0.001 | 0.120 |  |
| Trunk Muscle Mass (kg) | 25.10±2.98 | 22.88±1.73 | 27.39±2.14 | 25.16±3.04 | <0.001 | 1.361 |  |
| Leg Muscle Balance (%) | 0.08 (0.04, 0.15) | 0.08 (0.04, 0.14) | 0.09 (0.05, 0.17) | 0.08 (0.04, 0.15) | <0.001 | 0.096 |  |
| VFA (cm²) | 90.75 (73.38, 112.50) | 79.60 (66.00, 94.40) | 105.30 (87.53, 127.20) | 86.60 (70.60, 106.90) | <0.001 | 0.612 |  |
| SMI (kg/m²) | 7.90±0.70 | 7.41±0.46 | 8.40±0.52 | 7.94±0.73 | <0.001 | 1.210 |  |
| FFMI (kg/m²) | 18.52±1.64 | 17.44±1.11 | 19.63±1.31 | 18.62±1.69 | <0.001 | 1.097 |  |
| FMI (kg/m²) | 7.16±2.16 | 6.43±1.80 | 7.96±2.23 | 6.84±2.11 | <0.001 | 0.492 |  |
| InBody Score | 68.39±6.11 | 67.43±5.22 | 69.23±6.83 | 69.51±5.79 | <0.001 | 0.240 |  |
| BMC (kg) | 3.06±0.45 | 2.72±0.24 | 3.34±0.35 | 3.61±0.39 | <0.001 | 1.846 |  |
| TBW_LBM_Ratio | 73.58±0.25 | 73.64±0.22 | 73.51±0.26 | 73.61±0.18 | <0.001 | 0.386 |  |
| WC, (cm) | 93.09±9.76 | 87.46±6.72 | 98.99±8.96 | 92.79±9.58 | <0.001 | 0.923 |  |
| HC (cm) | 99.30±5.43 | 95.58±3.34 | 103.05±4.49 | 100.17±5.38 | <0.001 | 1.164 |  |
| WHR | 1691 (37.58) | 758 (35.34) | 846 (41.03) | 87 (29.69) | <0.001 | 0.159 |  |
| Hypertension, (*n*, %) | 810 (18.00) | 421 (19.63) | 359 (17.41) | 30 (10.24) | <0.001 | 0.177 |  |
| Diabetes (n, %) | 107 (2.38) | 53 (2.47) | 52 (2.52) | 2 (0.68) | 0.143 | 0.098 |  |
| CHD (*n*, %) | 2376 (52.80) | 818 (38.14) | 1414 (68.57) | 144 (49.15) | <0.001 | 0.422 |  |

Abbreviations: BMC, bone mineral content; BMI, body mass index; CHD, coronary heart disease; ECW, extracellular water; FMI, fat mass index; FFMI, fat-free mass index; HC, hip circumference; ICW, intracellular water; LBM, lean body mass; PBF, percent body fat; SMI, skeletal muscle index; SMM, skeletal muscle mass; TBW, total body water; TBW_LBM_Ratio, ratio of total body water to lean body mass; VFA, visceral fat area; WC, waist circumference; WHR, waist-to-hip ratio.

Table S3. Characteristics of Body Composition Cluster Profilesin females.

| Characteristic | Total (*N* =3446) | LML (*N* =1694) | HMO (*N* =1546) | FIHP (*N* =206) | *P* | SMD |  |
| --- | --- | --- | --- | --- | --- | --- | --- |
| Age (years) | 51.31±12.66 | 51.02±13.08 | 52.63±11.83 | 43.73±12.34 | <0.001 | 0.479 |  |
| Height (cm) | 160.02±5.57 | 158.59±5.23 | 161.64±5.44 | 159.67±5.91 | <0.001 | 0.370 |  |
| Weight (kg) | 59.86±8.68 | 53.60±4.43 | 66.82±6.72 | 59.10±8.58 | <0.001 | 1.376 |  |
| BMI (kg/m²) | 23.38±3.24 | 21.35±2.01 | 25.63±2.82 | 23.15±3.30 | <0.001 | 1.071 |  |
| TBW (L) | 28.91±3.15 | 26.90±2.15 | 31.11±2.54 | 28.89±3.11 | <0.001 | 1.105 |  |
| ICW (L) | 17.16±3.06 | 16.51±1.35 | 19.12±1.60 | 7.70±0.85 | <0.001 | 6.162 |  |
| ECW (L) | 11.55±2.02 | 10.39±0.82 | 11.99±0.97 | 17.81±1.94 | <0.001 | 3.524 |  |
| Protein Mass (kg) | 7.88±1.19 | 7.14±0.58 | 8.26±0.70 | 11.07±1.18 | <0.001 | 2.953 |  |
| Mineral Mass (kg) | 2.74±0.31 | 2.57±0.22 | 2.93±0.28 | 2.76±0.34 | <0.001 | 0.868 |  |
| Fat Mass (kg) | 20.52±5.99 | 16.99±3.86 | 24.51±5.41 | 19.61±5.89 | <0.001 | 0.999 |  |
| Muscle Mass (kg) | 37.06±4.04 | 34.48±2.77 | 39.88±3.27 | 37.07±4.00 | <0.001 | 1.102 |  |
| LBM (kg) | 39.32±4.29 | 36.61±2.93 | 42.30±3.48 | 39.29±4.33 | <0.001 | 1.087 |  |
| SMM (kg) | 21.16±2.56 | 19.54±1.77 | 22.93±2.08 | 21.23±2.54 | <0.001 | 1.089 |  |
| PBF (%) | 33.74±5.90 | 31.46±5.51 | 36.39±5.14 | 32.72±6.07 | <0.001 | 0.599 |  |
| Arm Muscle Balance (%) | 0.05 (0.02, 0.08) | 0.04 (0.02, 0.07) | 0.05 (0.02, 0.08) | 0.04 (0.02, 0.07) | <0.001 | 0.214 |  |
| Trunk Muscle Mass (kg) | 17.92±2.05 | 16.51±1.29 | 19.48±1.56 | 17.70±1.94 | <0.001 | 1.270 |  |
| Leg Muscle Balance (%) | 0.06 (0.03, 0.10) | 0.05 (0.02, 0.09) | 0.07 (0.03, 0.11) | 0.06 (0.03, 0.10) | <0.001 | 0.173 |  |
| VFA (cm²) | 98.90 (72.80, 126.30) | 78.60 (62.60, 101.18) | 122.80 (100.10, 145.10) | 91.10 (64.45, 115.10) | <0.001 | 0.870 |  |
| SMI (kg/m²) | 6.21±0.61 | 5.81±0.42 | 6.66±0.46 | 6.22±0.60 | <0.001 | 1.177 |  |
| FFMI (kg/m²) | 15.34±1.31 | 14.56±0.96 | 16.19±1.09 | 15.40±1.34 | <0.001 | 0.989 |  |
| FMI (kg/m²) | 8.04±2.39 | 6.80±1.70 | 9.44±2.26 | 7.74±2.42 | <0.001 | 0.832 |  |
| InBody Score | 68.83±4.87 | 69.85±4.09 | 67.60±5.40 | 69.73±4.45 | <0.001 | 0.310 |  |
| BMC (kg) | 2.30±0.30 | 2.13±0.19 | 2.42±0.24 | 2.89±0.27 | <0.001 | 2.144 |  |
| TBW_LBM_Ratio | 73.52±0.22 | 73.48±0.21 | 73.55±0.24 | 73.60±0.15 | <0.001 | 0.393 |  |
| WC, (cm) | 83.85±8.46 | 78.16±4.90 | 90.02±7.09 | 84.32±8.34 | <0.001 | 1.194 |  |
| HC (cm) | 92.93±4.89 | 89.43±2.75 | 96.57±3.88 | 94.41±4.63 | <0.001 | 1.312 |  |
| WHR | 0.90±0.05 | 0.87±0.04 | 0.93±0.05 | 0.88±0.05 | <0.001 | 0.837 |  |
| Hypertension, (*n*, %) | 737 (21.39) | 271 (16.00) | 426 (27.55) | 40 (19.42) | <0.001 | 0.188 |  |
| Diabetes (n, %) | 238 (6.91) | 83 (4.90) | 146 (9.44) | 9 (4.37) | <0.001 | 0.134 |  |
| CHD (*n*, %) | 16 (0.46) | 6 (0.35) | 10 (0.65) | 0 (0.00) | 0.402 | 0.080 |  |
| Age (years) | 786 (22.81) | 164 (9.68) | 586 (37.90) | 36 (17.48) | <0.001 | 0.467 |  |

Abbreviations: BMC, bone mineral content; BMI, body mass index; CHD, coronary heart disease; ECW, extracellular water; FMI, fat mass index; FFMI, fat-free mass index; HC, hip circumference; ICW, intracellular water; LBM, lean body mass; PBF, percent body fat; SMI, skeletal muscle index; SMM, skeletal muscle mass; TBW, total body water; TBW_LBM_Ratio, ratio of total body water to lean body mass; VFA, visceral fat area; WC, waist circumference; WHR, waist-to-hip ratio.

Table S4 Consensus Top 15 Critical Features of MASLD Identified by Boruta, XGBoost, and RF Algorithms

| Rank | Feature | Ensemble Score | Selected_By | Boruta Rank | XGBoost Rank | RF Rank | Consensus Level |
| --- | --- | --- | --- | --- | --- | --- | --- |
| 1 | TG | 0.21744 | Boruta; XGBoost; RF | 1 | 25 | 25 | 3 |
| 2 | ALT | 0.204062 | Boruta; XGBoost; RF | 1 | 29 | 29 | 3 |
| 3 | GGT | 0.200272 | XGBoost; RF | 1 | 45 | 45 | 2 |
| 4 | HDLC | 0.197695 | Boruta; XGBoost; RF | 1 | 12 | 12 | 3 |
| 5 | UA | 0.194576 | XGBoost; RF | 1 | 50 | 50 | 2 |
| 6 | GLU | 0.189108 | XGBoost; RF | 1 | 34 | 34 | 2 |
| 7 | ChE | 0.188914 | Boruta; XGBoost; RF | 1 | 10 | 10 | 3 |
| 8 | HbA1c | 0.183966 | XGBoost; RF | 1 | 37 | 37 | 2 |
| 9 | SBP | 0.181619 | XGBoost; RF | 1 | 47 | 47 | 2 |
| 10 | DBP | 0.180779 | Boruta; XGBoost; RF | 1 | 18 | 18 | 3 |
| 11 | FT3 | 0.179347 | Boruta; XGBoost; RF | 1 | 16 | 16 | 3 |
| 12 | HCT | 0.179068 | Boruta; RF | 1 | 8 | 8 | 2 |
| 13 | FT4 | 0.178917 | Boruta; RF | 1 | 19 | 19 | 2 |
| 14 | Age | 0.178629 | Boruta; XGBoost | 1 | 26 | 26 | 2 |
| 15 | LymphCount | 0.178491 | Boruta; XGBoost | 1 | 13 | 13 | 2 |

The “Ensemble Score” represents a composite metric derived from the rankings and selections made by Boruta, XGBoost, and RF.

Table S5. Variance Inflation Factor (VIF) analysis for candidate predictors.

| Feature | GVIF | Df | GVIF^(1/(2*Df)) |
| --- | --- | --- | --- |
| cluster | 1.135 | 2 | 1.032 |
| Age | 1.432 | 1 | 1.197 |
| SBP | 2.733 | 1 | 1.653 |
| DBP | 2.698 | 1 | 1.642 |
| UA | 1.438 | 1 | 1.199 |
| ALT | 1.325 | 1 | 1.151 |
| GGT | 1.423 | 1 | 1.193 |
| ChE | 1.034 | 1 | 1.017 |
| TG | 1.286 | 1 | 1.134 |
| HDLC | 1.285 | 1 | 1.134 |
| HbA1c | 3.440 | 1 | 1.855 |
| GLU | 3.317 | 1 | 1.821 |
| FT4 | 1.106 | 1 | 1.052 |
| FT3 | 1.263 | 1 | 1.124 |
| HCT | 1.457 | 1 | 1.207 |
| LymphCount | 1.112 | 1 | 1.054 |

Abbreviations: ALT, alanine aminotransferase; ChE, cholinesterase; DBP, diastolic blood pressure; FT3, free triiodothyronine; FT4, free thyroxine; GGT, gamma-glutamyl transferase; GLU, glucose; HCT, hematocrit; HbA1c, hemoglobin A1c; HDLC, high-density lipoprotein cholesterol; LymphCount, lymphocyte count; SBP, systolic blood pressure; TG, triglycerides; UA, uric acid

Table S6. Internal validation of MASLD prediction models: hold-out and bootstrap-corrected discrimination (B = 300).

| Model Specification | Hold-out AUC (30% test) | Apparent AUC (full data) | Optimism | Corrected AUC |
| --- | --- | --- | --- | --- |
| Model 1 (Crude) | 0.655 | 0.6548 | 0.0003 | 0.6545 |
| Model 2 (Adjusted) | 0.749 | 0.7495 | 0.0004 | 0.7490 |
| Model 3 (Full) | 0.863 | 0.8670 | 0.0012 | 0.8658 |

Hold-out AUC: performance on the independent 30% test set (never used in model development). Apparent AUC: performance on the full dataset before correction. Optimism = Apparent AUC − Corrected AUC, estimated via 300 bootstrap replications. Corrected AUC = Apparent AUC − Optimism.

Fig S1. UMAP parameter optimization in male participants: Shepard Goodness vs. Trustworthiness across n_neighbors values.

Fig S2. UMAP parameter optimization in female participants: Shepard Goodness vs. Trustworthiness across n_neighbors values.

Fig S3. Distribution of body composition characteristics across UMAP-derived phenotypes in males. The distributions were color-coded for LML, HMO, and FIHP clusters.

Fig S4. Distribution of body composition characteristics across UMAP-derived phenotypes in females. The distributions were color-coded for LML, HMO, and FIHP clusters.
